# Tumor-Derived Exosomes and Their Role in Breast Cancer Metastasis

**DOI:** 10.3390/ijms232213993

**Published:** 2022-11-13

**Authors:** Shaojuan Huang, Ming Dong, Qiang Chen

**Affiliations:** 1 Cancer Center, Faculty of Health Sciences, University of Macau, Taipa, Macau SAR, China; 2 Guangzhou Laboratory, Guangzhou 510005, China; 3 MOE Frontier Science Centre for Precision Oncology, University of Macau, Taipa, Macau SAR, China

**Keywords:** breast cancer, metastasis, exosome, extracellular vesicles, organotropism

## Abstract

Breast cancer has been the most common cancer in women worldwide, and metastasis is the leading cause of death from breast cancer. Even though the study of breast cancer metastasis has been extensively carried out, the molecular mechanism is still not fully understood, and diagnosis and prognosis need to be improved. Breast cancer metastasis is a complicated process involving multiple physiological changes, and lung, brain, bone and liver are the main metastatic targets. Exosomes are membrane-bound extracellular vesicles that contain secreted cellular constitutes. The biogenesis and functions of exosomes in cancer have been intensively studied, and mounting studies have indicated that exosomes play a crucial role in cancer metastasis. In this review, we summarize recent findings on the role of breast cancer-derived exosomes in metastasis organotropism and discuss the potential promising clinical applications of targeting exosomes as novel strategies for breast cancer diagnosis and therapy.

## 1. Introduction

Breast cancer is a common frequently occurring malignancy among women worldwide, and it has surpassed lung cancer to become the most diagnosed cancer all over the world with around 2.3 million new cases, accounting for 11.7% of all cancer cases and 24.5% of female cancers [1]. With recent advances in early diagnosis and therapeutic strategies including neoadjuvant therapy, endocrine therapy, molecular targeted therapy, and immunotherapy [2,3], the prognosis of breast cancer has greatly improved. However, breast cancer patients with distant metastasis have worse outcomes, and the five-year survival rate was less than 30% [4]. With approximately 685,000 deaths in 2020, it remains the first leading cause of cancer death in women [1]. Therefore, there is an urgent need to understand the molecular mechanisms underlying breast cancer metastasis for developing novel therapeutic strategies. Exosomes, as one type of extracellular vesicle (EVs), have been reported to play a crucial role in cancer metastasis, namely, contributing to form pre-metastatic niches, influence the tumor microenvironment, and identify specific organotropic metastasis. Here, we endeavor to highlight the role of tumor-derived exosomes in breast cancer metastasis, elucidate the underlying mechanism of metastasis organotropism mediated by exosomes, and prospect the potential application of exosomes in breast cancer therapeutics.

### 1.1. Breast Cancer Classification

Breast cancer develops from epithelial cells in the terminal duct lobular units and can be classified into two subtypes histologically, including ductal carcinoma in situ (DCIS) and invasive ductal carcinoma (IDC) [5,6]. According to the expression of estrogen receptor (ER), progesterone receptor (PR), human epidermal growth factor receptor 2 (HER2), and Ki-67 labelling index (Ki-67) which reflects the proliferation [7], breast cancer has four primary molecular subtypes, namely luminal A, luminal B, HER2-positive, and triple-negative breast cancer (TNBC) [5,8]. The luminal A subtype is ER/PR-positive, with lower levels of Ki-67 (<14), accounting for about 60–70% of diagnostic breast cancers. The luminal B subtype is ER-positive combined with HER-2 positive or Ki-67 high (≥14), accounting for about 10–20%. Both luminal A and luminal B breast cancer are likely to benefit from endocrine therapy, and patients with luminal A breast cancer have a better prognosis compared to luminal B. HER2-positive subtype is ER/PR-negative and HER2-positive, with a diagnostic rate of approximately 13−15%. This subtype can benefit from treatment targeted to HER2 and chemotherapy with good prognosis. TNBC is characterized by ER, PR, and HER2 negativity in 10−15% of cases. Breast cancer susceptibility gene 1 (*BRCA1*) is a major breast cancer suppressor gene that encodes a protein critical for maintaining genome stability; its mutation predisposes women to TNBC [9]. As *BRCA1* mutation impairs homologous recombination repair, poly (ADP-ribose) polymerase (PARP) inhibitors have been approved as target therapy for metastatic TNBC [10]. However, due to its highly aggressive clinical properties, TNBC still has a poorer prognosis compared to other breast cancer subtypes [2,5,11,12]. Although the five-year survival rate for women diagnosed with breast cancer exceeds 90%, all breast cancer subtypes have the potential to exhibit adverse clinic features, such as high invasiveness and recurrence, mainly caused by metastasis [5].

### 1.2. Breast Cancer Metastasis

**Figure 1:**
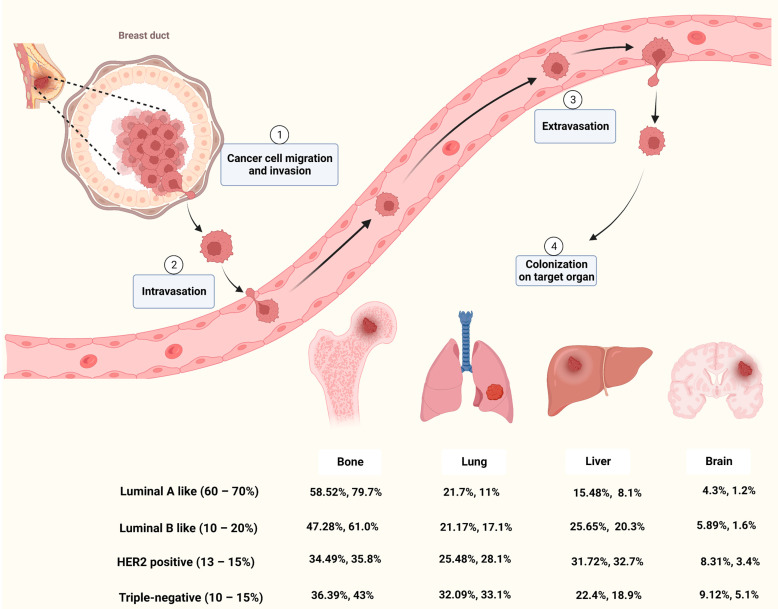
Breast cancer metastasis to bone, lung, liver and brain [5,15,16]. Figure is created by BioRender.

Metastasis occurs frequently and accounts for as much as 90% of cancer-related deaths [13]. Breast cancer metastasis is also frequently diagnosed and exhibits organ tropism to lung, brain, bone and liver, which is highly heterogeneous and affects treatment outcomes and patient prognosis [14]. As shown in Figure 1, breast cancer patients are most prone to bone metastasis, accounting for 50.7−68.8% of all metastatic cases with different molecular subtypes. The lung and liver were similar, with 16.0−23.9% and 13.3−19.7% of breast cancer metastasis occurring in the lung or liver, respectively. The incidence of brain metastasis is approximately 1.9−5.7% of all metastatic cases. Based on molecular subtypes, lung metastasis most commonly occurs in TNBC, accounting for about 32% of patients with metastasis, while the HER2-positive subtype is likely to have liver metastasis [15,16].

Cancer metastasis is a complicated process that goes through multiple steps such as invasion, intravasation, extravasation and colonization on target organ (Figure 1). Metastasis of tumor cells to distant organs requires not only tumor cell invasiveness, but also a microenvironment that is conducive to tumor survival and proliferation in secondary organs [17]. Although the molecular mechanisms of breast cancer metastasis are not fully understood, mounting studies have shown that primary cancer cells could secrete factors to remodel the microenvironment of the target organ and prime it into a site favorable for cancer cell proliferation, known as pre-metastatic niche (PMN) [18,19,20]. Among these factors, EVs secreted by cancer cells play important roles in microenvironment remodeling and metastatic organotropism [21].

### 1.3. EVs and Exosome

EVs are membrane-bound vesicles secreted by various cells which play critical roles in cell-cell communication under physiological and pathological conditions [22]. In general, EVs could be divided into three types based on their morphology: exosomes (30–150 nm), micro-vesicles (150–1000 nm) and apoptotic bodies (500–2000 nm) [23]. Nowadays, EVs are considered non-negligible factors in cellular homeostasis and mediators of cancer metastasis [24]. Exosomes are small EVs with phospholipid bilayers whose heterogeneous “cargoes”, such as protein, lipids, RNA and DNA, vary from different types of cells [25,26]. These cargoes are located inside or on the surface of exosomes and mediate the communication between original cells and recipient cells [26].

Exosomes are generated originally from early endosomes by fusion of endocytic vesicles with plasma membranes. Early endosomes subsequently mature into late endosomes and form the multivesicular bodies (MVBs) containing intraluminal vesicles (ILVs). MVBs could either fuse with plasma membrane for exocytosis of contained ILVs (namely exosomes) into the extracellular space, or with lysosomes or autophagosomes for degradation [27,28]. ILV formation is largely dependent on the endosomal sorting complex required for transport (ESCRT) function. ESCRT is an intricate machinery which is made up of five complexes, which include the ESCRT-0, -Ⅰ, -Ⅱ, -Ⅲ and Vps4-Vta1 complexes [27,29]. Beside the ESCRT-dependent pathway, an alternative pathway could also be involved in exosome biogenesis. Proteolipid proteins (PLP) could be incorporated into ILVs in a sphingolipid ceramide-dependent manner. Then, ceramide induces raft-based microdomains formation and coalescence and promotes the budding of ILVs [30]. Moreover, tetraspanins, such as CD63, CD81, CD9, could regulate ESCRT-independent sorting [27]. Therefore, ESCRT dependent and independent mechanisms co-exist and function synergistically in exosome formation.

In recent years, studies have found that cancer-derived exosome could promote the progression of cancer, including cancer metastasis and drug resistance [31]. Exosomes have been proved to be involved in various processes of cancer metastasis, such as vascular leakiness, epithelial-mesenchymal transition (EMT) induction, immune escape and PMN formation [24,32,33]. Integrins are cell surface adhesion molecules that could mediate cell signaling by interacting with components of the extracellular matrix [34]. It has been demonstrated that integrins on exosomes could guide them into different organs, thereby inducing PMN formation and promoting cancer metastasis. Exosomes with integrins α_6_β_4_ and α_6_β_1_ tend to accumulate in the lung, while integrin αvβ5 preferably drives exosomes into the liver [35]. In addition, comparative proteomics of exosomes derived from different breast cancer cells demonstrated that their exosomal proteins are heterogenous, which is associated with different cancer cell metastatic properties [36]. These studies suggest that exosomes play a crucial role in causing metastasis organotropism through various mechanisms. In the following section, we will summarize the contribution of exosomes to the tropism of breast cancer metastasis to different organs, namely bone, lung, liver and brain, and the metastatic models used in these findings (Table 1), which will help to comprehensively decipher how exosomes are involve in breast cancer metastasis. 

## 2. Exosomes and Breast Cancer Metastasis Organotropism

### 2.1. Exosomes Mediate Breast Cancer Metastasis to Bone

Bone is the most likely site for all types of breast cancer to metastasize. Patients with bone metastasis are often accompanied by other serious complications, such as severe bone pain, fractures, serious hypercalcemia, and nerve compression syndromes, which seriously affect the patients’ life expectancy and quality of life [66]. Bone metastasis involves a complicated interaction between cancer cells and bone microenvironments. Under normal circumstances, bone undergoes a dynamic balance of bone resorption and bone formation, mediated by osteoclasts and osteoblasts, respectively, while bone metastasis often exhibits a disordered balance of this process [67]. Therefore, there are mainly two types of bone metastasis, osteolytic and osteoblastic, depending on which cells are overactive [68]. Osteolytic bone metastasis frequently occurs in breast cancer, mainly due to the activation of the RANK-RANKL signaling pathway that mediates osteoclastogenesis. Breast cancer stimulates RANKL expression in osteoblasts by producing parathyroid hormone-related protein (PTHrP), which in turn leads to excessive osteolysis and promotes bone metastasis by activating RANK-RANKL signaling [66,67,69]. Besides PTHrP, many other factors such as calcium-sensing receptor (CaSR), TNF-α, and interleukins also affect bone metastasis [67].

**Figure 2:**
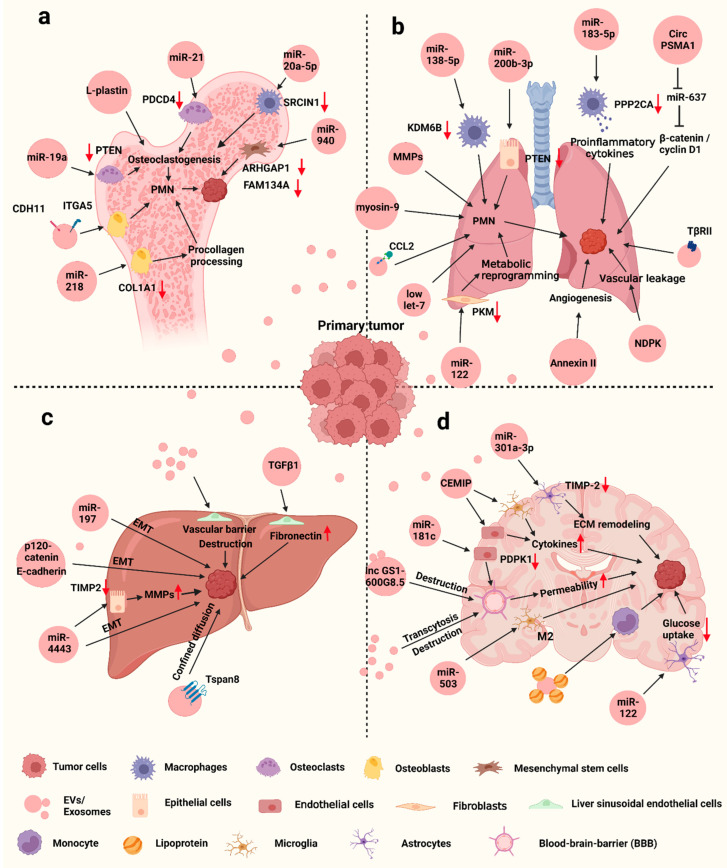
Exosomes mediate breast cancer metastasis to bone (**a**), lung (**b**), liver (**c**) and brain (**d**). Figure is created by BioRender.

Furthermore, recent studies have shown that EVs, especially exosomes, play an important role in bone metastasis of breast cancer (Figure 2a). The main role is that exosomes can bring osteoclastogenesis-enhancing miRNA into bone, thereby promoting osteolytic metastasis. As an osteolytic phenotype-inducing cell, MDA-MB-231 could facilitate the osteogenic differentiation of mesenchymal stem cells via exosomal miR-940 targeting Rho GTPase activating protein 1 (ARHGAP1) and FAM134A [37]. MDA-MB-231 also secreted exosomal miR-218 to impede procollagen processing during osteoblast differentiation and tip the balance toward osteolysis to form a metastatic bone niche [38]. Guo et al. found that MDA-MB-231-derived exosomes could transfer miR-20a-5p to bone marrow macrophages, which then enhanced osteoclast proliferation and differentiation by targeting SRC Kinase Signaling Inhibitor 1 (SRCIN1) [39]. It is also reported that exosomal miR-21 produced from MDA-MB-231 derived cells with high bone metastatic ability could contribute to bone lesion and PMN formation via targeting programmed cell death 4 (PDCD4), which has an inhibitory function on osteoclast differentiation [40]. Unlike MDA-MB-231 cells, ER^+^ bone-tropic breast cancer cells could produce exosomal miR-19a, which promotes osteoclastogenesis and bone metastasis by suppressing PTEN expression and inducing NF-κB and AKT pathways [41].

Besides miRNAs, exosomes can also carry other “cargoes” to facilitate bone metastasis. L-plastin was found to be a soluble factor secreted from MDA-MB-231 cells, belonging to the actin-binding proteins family. L-plastin could be encapsulated in exosomes, which stimulated osteoclastogenesis and further promoted bone metastasis [42]. Breast cancer cells with high runt-related transcription factor 2 (RUNX2) expression could secrete EVs with high levels of cadherin 11 (CDH11) and integrin α5 (ITGA5), which synergistically promote osteogenic PMN formation. Mechanistically, CDH11 mediated tumor-derived EVs uptake by osteoblasts and ITGA5 was responsible for the formation of PMN that facilitated cancer cell colonization in bone [43]. Integrin-binding Sialoprotein (IBSP) secreted by ER^+^ bone-tropic breast cancer cells could bind to αvβ3 integrin and attract osteoclasts, assisting the delivery of exosomal miR-19a to osteoclast to induce bone metastatic lesions [41].

### 2.2. Exosomes Mediate Breast Cancer Metastasis to Lung

Lung is another target of frequent breast cancer metastasis. Pulmonary capillaries are composed of endothelial cells surrounded by a based membrane and adjacent alveolar cells. Tumor cells need to adhere to this endothelial membrane and extravasate into lung parenchyma to establish metastatic tumors [70]. The process of lung metastasis is affected by many factors, among which exosomes play a critical role in remodeling the immune microenvironment and inducing EMT (Figure 2b).

Tumor-derived exosomes promote lung metastasis via non-coding RNAs that mediate relevant signaling. Exosomal miR-122 was secreted by high metastatic breast cancer cells and increased nutrient availability in lung metastatic cancer cells by downregulating glycolytic enzyme pyruvate kinase (PKM) in lung fibroblasts [44]. Recent study further found that exosomal miR-122 could also target PKM in pancreatic β-cells to suppress insulin secretion and disrupt systemic glucose homeostasis to promote cancer progression [71]. Besides metabolism reprogramming in metastatic organ, exosome also mediated miRNA transfer from cancer cells to other cells for microenvironment remodeling and promoting the pre-metastatic niche formation in the lung. Exosomal miR-138-5p and miR-183-5p secreted by cancer cells could modulate tumor-associated macrophages’ (TAMs) activity to enhance lung metastasis via targeting KDM6B and PPP2CA, respectively [45,46]. miR-200b-3p was enriched in tumor-derived exosomes and transferred to lung, thereby increasing the expression of C-C motif chemokine ligand 2 (CCL2) by targeting PTEN. CCL2 could further recruit myeloid-derived suppressor cells (MDSC) and contribute to the establishment of an immunosuppressive microenvironment for metastasis [47]. However, exosomes that could enhance metastasis are not always enriched with miRNA. Lin28 is an RNA-binding protein that regulates the expression of miRNA let-7 family members and acts as a modulator for self-renewal of embryonic stem cells [72,73]. Lin28B is mainly expressed in TNBC and could promote breast cancer progression [74]. Recent studies indicate that Lin28B could increase breast cancer stem cell population, a major source of low let-7 exosomes. Moreover, these exosomes contribute to neutrophil N2 transformation and induce immunosuppressive PMN, promoting lung metastasis [48]. In addition to miRNA, tumor-derived exosomal long non-coding RNA (lncRNA) and circular RNA (CircRNA) also contribute to lung metastasis of breast cancer. Abnormal expression of lncRNAs in exosomes facilitated the formation of a lung metastatic microenvironment [75]. Exosomal circPSMA1 could act as a “sponge” to neutralize miR-637, thereby upregulating the expression of Akt1 and affecting downstream genes such as β-catenin and cyclin D1. The circPAMA1/miR637/Akt1/β-catenin (cyclin D1) axis promoted not only tumorigenesis, but also TNBC metastasis to the lung [49].

Some protein factors could also be the cargo in exosomes and promote metastasis. Recent studies found that cytokines in a tumor microenvironment can regulate the organotropism of metastasis via exosome secretion. CCL2 could directly bind to tumor-derived exosomes through the glycosaminoglycan side chains of proteoglycans. Furthermore, these CCL2-decorated exosomes were directed to CCR2-expressing cells, especially in the lung, leading to the lung metastasis burden [50]. Another chemokine, CCL5 expressed by tumor cells not only induces macrophage recruitment, but also promotes the secretion of more EVs by tumor cells, whereby EVs educated macrophages into TAMs and enhanced metastasis in the lung [76]. Besides cytokines, tumor-derived exosomes carry other proteins to contribute to lung metastasis. TGF-β type II receptor (TβRII) could be transferred by exosomes from malignant cells and activate TGF-β signaling in recipient cells. On the one hand, exosomal TβRII could induce TGF-β activation to initiate EMT in low-grade cancer cells, thus enhancing cancer stemness and metastasis. On the other hand, exosomal TβRII could also induce CD8^+^ T cell exhaustion by activating SMAD3, thereby leading to immunosuppression [51]. Feng et al. found that macrophage infiltration was positively correlated with the levels of signal-induced proliferation-associated 1(SIPA1) in invasive breast ductal carcinoma. SIPA1 could increase the level of myosin-9 in exosomes, which enhances macrophage infiltration and lung metastasis [52]. Aspartate β-hydroxylase (ASPH) could induce Notch signaling activation in breast cancers and orchestrate pro-oncogenic/pro-invasive cargoes into tumor-derived exosomes, especially matrix metalloproteinases (MMPs). These exosomal cargoes could enhance breast cancer metastasis in distant organs, especially the lung [53]. Furthermore, exosomal MMP-1 generated by high metastatic cells could mediate EMT in low metastatic cells by interacting with membrane G protein receptor protease activated receptor 1 (PAR1) and enhance their migration and invasion [54]. Exosome could also carry nucleoside diphosphate kinase A and B (NDPK) and Annexin II, thereby enhancing lung metastasis by promoting the vascular leakage and angiogenesis in the lung, respectively [55,56].

### 2.3. Exosomes Mediate Breast Cancer Metastasis to Liver

As mentioned above, liver metastasis is also an important subgroup of breast cancer metastasis diagnosed, often occurring in HER2-positive breast cancer with poor prognosis [15,16]. The microenvironment in both primary tumors and liver tissues undergoes great changes during the establishment of secondary metastatic sites. Many factors could be involved in breast cancer liver metastasis, such as inflammatory factors, chemokines and the related receptors, and cell adhesion molecules, etc., which could promote the establishment of a pro-inflammatory environment and EMT in tumors. In addition, there are some factors related to liver microenvironment that affect metastasis, such as angiogenesis-related factors, hypoxia-regulated genes, liver metabolic status, and the interaction between sinusoidal capillaries and cancer cells [77,78]. Recent studies indicate that exosomes are also extensively involved in liver metastasis (Figure 2c). Similarly, exosomes could contribute to liver metastasis by carrying miRNAs against the relevant targets. Highly invasive breast cancer cells secreted exosomes with high level of miR-4443 against tissue inhibitors of metalloproteinase 2 (TIMP2); the exosomes mainly accumulated in liver to upregulate MMP-2 and induced liver metastasis [57]. Exosomal miR-197 was produced by enriched breast cancer stem cells and downregulated the expression of PPARγ, thereby activating EMT in cancer cells and facilitating liver metastasis [58]. Tetraspanins are transmembrane proteins associated with cell membrane compartmentalization [79]. Tspan8, a member of tetraspanins, enhanced breast cancer metastasis to the liver. Mechanistically, Tspan8 promoted the secretion of EVs carrying high levels of E-cadherin and p120-catenin, thereby increasing liver metastases by modulating the EMT-MET programme [59]. One recent study indicated that Tspan8 not only enhances exosome secretion, but also promotes the uptake of exosome in some tissues, including liver, through confined diffusion, thus promoting tumor progression [80]. With the development of new technologies, more molecular mechanisms by which exosomes are involved in liver metastasis have been discovered. Based on a three-dimensional microfluidic liver chip, Kim et al. found that breast cancer-derived exosomes activated liver sinusoidal endothelial cells (LSECs), leading to endothelial-to-mesenchymal transition and disruption of the vascular barrier. Furthermore, exosomes upregulated fibronectin on LSECs through delivering TGFβ1, which facilitates cancer cell attachment to the liver microenvironment [60].

### 2.4. Exosome Mediate Breast Cancer Metastasis to Brain

Unlike other tissues, brain metastasis is a heterogeneous process involving the interaction between the tumor cell and the blood-brain barrier (BBB). The BBB is mainly composed of astrocytes, pericytes and endothelial cells, which make up the neurovascular unit. Endothelial cells are the first to crosstalk with circulating tumor cells [81]. Under normal conditions, BBB controls the supply of essential nutrients to brain cells, protects the brain from toxic compounds in the blood, and filters the harmful factors into the blood, which plays an important role in maintaining central nervous system homeostasis [82]. Although BBB also acts as a barrier against circulating tumor cell (CTCs) infiltration, many studies have revealed that the changes in molecular and cellular signal pathways are involved in this process to disrupt the BBB and establish the PMN for brain metastasis [83]. Meanwhile, exosomes also participate in the process through multiple mechanisms (Figure 2d). According to single cell force spectroscopy by atomic force microscopy, exosomes from cancer cells reduced brain endothelial adhesion when in direct contact with breast cancer cells, suggesting that exosomes may modulate the adhesiveness of brain endothelium and affect their permeability [84]. Tubulin tyrosine ligase-like 4 (TTLL4) is a cytoskeleton-associated protein, and its expression was positively correlated with brain metastasis. Mechanistically, upregulated TTLL4 increased β-tubulin glutamylation and MVB trafficking, which increased adhesion of breast cancer cells to BBB endothelial cells as well as permeability of these endothelial cells by altering exosome signatures [85]. It suggests that cancer-derived exosomes could enhance brain metastasis by disrupting the BBB through certain factors. miR-181c could be enriched in brain metastatic breast cancer cell-derived exosomes and target 3-phosphoinositide-dependent protein kinase-1(PDPK1) in endothelial cells. Downregulation of PDPK1 further reduced phosphorylation of cofilin and led to abnormal actin filament organization, thereby destroying BBB and promoting brain metastasis [61]. Cancer-derived exosomes also transferred lncRNA GS1-600G8.5 to endothelial cells and increased the permeability of BBB to enhance the passage of cancer cells through the BBB. However, the downstream targets of this lncRNA are unclear [62].

Recent studies have shown that exosome could also remodel a brain microenvironment that favors cancer cell colonization and proliferation. Cell migration-inducing and hyaluronan-binding protein (CEMIP), a Wnt-signaling associated protein, was identified as a dominant exosomal protein in brain metastatic cells, with low or undetectable levels in exosomes from lung and bone metastatic cells. CEMIP-positive exosomes were taken up by brain endothelial and microglial cells and contribute to the establishment of PMN by causing the cerebral vascular remodeling through upregulation of pro-inflammatory cytokines [63]. LncRNA X-inactive-specific transcript (XIST) was found to be significantly downregulated in brain metastatic tumors, and its expression was negatively correlated with brain metastasis. Besides enhancing aggressiveness of cancer cells by induction of EMT and c-Met signaling, loss of XIST could increase exosomal miR-503 secretion, which triggered M2 polarization in microglia and suppressed T cell proliferation to form PMN [64]. In addition, miR-301a-3p enriched exosomes were taken up by astrocytes via non-canonical Cdc42-dependent endocytosis, and these exosomes resulted in extracellular matrix remodeling by suppressing TIMP-2 expression in preparation for a metastasis microenvironment [65]. To enhance the effect of tumor-derived exosomes on brain metastasis, exosomes can manipulate the brain endothelial cells to facilitate their transfer into brain parenchyma by downregulating the expression of Rab7 [86] and can also bind with low-density lipoprotein (LDL) during circulation, causing the LDL aggregation and promoting monocyte uptake [87].

In conclusion, tumor-derived exosomes contribute to breast cancer metastasis to bone, lung, liver and brain through multiple mechanisms, which provide potential therapeutic targets for diagnosis of and therapy for this deadly disease.

## 3. Clinical Application

### 3.1. Exosomes as Diagnostic Biomarkers for Breast Cancer

With the convenience of being non-invasive and highly efficient, liquid biopsy brings an opportunity for cancer diagnosis with detection through various body fluids such as blood or urine, rather than invasive methods to remove a piece of cancerous tissue [88]. Liquid biopsy has made some progress in establishing the diagnosis of various cancers by using certain molecules as biomarkers, including CTCs, circulating tumor DNA (ctDNA), tumor-educated platelet (TEP) and exosomes [89,90]. As mentioned above, exosomes contain specific “cargoes” derived from metastatic cancer cells, which not only play an important role in breast cancer progression and metastasis but may also have the potential to be biomarkers for diagnosing metastasis. To discover exosome-based biomarkers, Wang et al. established a comprehensive database—ExoBCD—by combining four high-throughput datasets, transcriptome of 1191 TCGA cases and manual mining of 950 studies. The database identified 306 valuable exosomal molecules, including 49 potential biomarkers and 257 biologically interesting molecules [91]. Since traditional detection methods, such as real-time PCR and Western Blotting analysis, are time-consuming and laborious and require exosome enrichment, which make them unsuitable for exosome-based diagnosis, it is necessary to develop alternative methods. A rapid, sensitive, and low-cost thermophoretic aptasensor (TAS) was developed for the analysis of cancer-associated protein profiles of plasma EVs. Based on this analysis, the EV protein signature was established and used to accurately monitor and predict metastatic breast cancer [92]. Kwizera et al. developed an inexpensive and highly efficient device based on the surface-enhanced Raman scattering (SERS) to detect exosomes and exosomal protein profiles. Using this device, they identified exosomal HER2 and EpCAM as biomarkers in the plasma of HER2-positive breast cancer patients [93]. Then, Lee et al. established another SERS-based platform to detect and quantify exosomal miRNAs in serum for breast cancer diagnosis [94]. In addition, a nano-sized fluorescent oligonucleotide probe-molecular beacon has also been developed for the measurement of miRNAs in blood exosomes with high sensitivity and specificity, such as miR-21 [95,96], miR-27a, miR-375 [96], and miR-1246 [97]. Recently, a microfluidic chip-based exosomal mRNA sensor was developed to directly detect exosomal ERBB2 in blood for the diagnosis of HER2-positive breast cancer [98]. Therefore, technological advances will enable exosomes to be used as biomarkers for breast cancer diagnosis in the future.

### 3.2. Engineered Exosomes for Therapeutics of Breast Cancer

The natural characteristics of exosomes, such as low toxicity, low immunogenicity, high-flexibility engineering, and inherent targeting and interaction with recipient cells, make them ideal drug carriers for breast cancer therapy [99,100,101,102]. First, exosomes can serve as delivery vesicles for chemotherapeutic drugs such as doxorubicin, with some engineering modification on their surface to improve their targeting efficiency and reduce side effects [103]. Hydrophobic drugs, such as Aspirin, could also be loaded into exosomes to increase their solubility and enhance their cytotoxicity against cancer cells [104]. In addition, to enhance the efficacy of PARP inhibitors, exosomes isolated from TNBC cells were loaded with Olaparib (PARP inhibitor) and superparamagnetic iron oxide (SPIO) nanoparticles, which could be tracked by magnetic particle imaging (MPI) and also effectively inhibited tumor growth [105]. Second, functional small RNAs, such as siRNA and miRNA, can be packaged into exosomes and delivered to cancer cells to downregulate target genes, thereby inhibiting cancer progression [106,107,108,109]. Third, engineered exosomes can be used as vaccines to stimulate an immune response against tumor cells. A novel exosome-like nanoparticles was developed from fibroblast activation protein-α (FAP) engineered cancer cells as a tumor vaccine, which induced robust and specific cytotoxic T lymphocyte immunity against tumor cells and reprogrammed the immunosuppressive microenvironment [110]. Exosomes from α-lactalbumin overexpressing breast cancer cells were packaged together with immunogenic cell death inducers—human neutrophil elastase (ELANE) and Hiltonol (a TLR3 agonist) to construct a vaccine, thereby priming dendritic cells in situ and improving subsequent tumor-reactive CD8+ T cell responses [111]. Exosome can also be engineered to display both anti-CD3 and anti-HER2 antibodies to mediate cytotoxic T cells that directly target HER2-positive breast cancer and improve immunotherapy [112]. Fourth, exosomes can be utilized as nanocarriers to provide cancer-targeted sonosensitizers for sonodynamic therapy (SDT), which employs reactive oxygen species (ROS) generated by ultrasonic excitation to kill cancer cells [113]. Indocyanine green-loaded exosomes were surface-modified with cancer-binding ligand to increase their target specificity, resulting in greater SDT against cancer cells [114]. Sinoporphyrin sodium (DVDMs) could also be loaded into tumor-derived exosomes to increase its efficiency in SDT [115]. Exosomes can be used not only for primary tumor therapy, but also be engineered to treat breast cancer metastasis. As exosomes derived from metastatic breast cancers have natural organotropism to lung [35] and brain [86], therapeutic drugs can be encapsulated with exosomes to target the relevant metastatic foci. Gold nanorods, a nanomaterial for photothermal therapy, were loaded into lung metastatic cancer cells-derived exosomes, exhibiting better therapeutic effects on lung metastases [116]. Finally, more engineered exosomes will be developed to improve precision therapy for breast cancers, especially with regard to metastasis.

## 4. Conclusions

Extensive studies have been conducted on the function of exosomes in breast cancer metastasis, and these studies have shown that exosomes can broadly affect its metastasis. By controlling the contents encapsulated in exosomes, cancer cells can remodel the microenvironment of their preferred distant organs through exosome secretion, further promoting the metastatic process of cancer and generating metastasis organotropism. Exosomes will become new cancer diagnostic markers in cancer therapy due to their own characteristics. Therefore, a comprehensive understanding of exosome functions in breast cancer metastasis could provide some new insights into their clinical applications.

## Figures and Tables

**Table 1:** Breast cancer models used to study the mechanism of exosomes promoting metastasis.

Metastatic Organs	Exosomal Molecules	Cell Lines	Metastasis Mouse Model	Reference
Bone	miR-940	MDA-MB-231	Calvaria implantation	[37]
	miR-218	MDA-MB-231, MCF-7	Tail vein injection of EVs	[38]
	miR-20a-5p	MDA-MB-231, MCF-7	In vitro	[39]
	miR-21	MDA-MB-231	Orthotopic model and Caudal artery injection	[40]
	miR-19a, IBSP	MDA-MB-231, MCF7, T47D	Intra-cardiac model, intratibial implantation and orthotopic model	[41]
	L-plastin	MDA-MB-231	Intratibial implantation	[42]
	CDH11, ITGA5	MDA-MB-231, 4T1, MCF7	Intra-cardiac model and orthotopic model	[43]
lung	miR-122	MDA-MB-231, MCF10DCIS.com	Intra-cardiac model and orthotopic model	[44]
	miR-138-5p	4T1	Tail vein injection	[45]
	miR-183-5p	4T1	Orthotopic model	[46]
	miR-200b-3p	4T1	Orthotopic model	[47]
	Let-7	4TO7	Orthotopic model, tail vein injection and intra-cardiac model	[48]
	circPSMA1	MDA-MB-231	Orthotopic model	[49]
	CCL2	EO771	Tail vein injection	[50]
	TβRII	MDA-MB-231, 4T1, 4T07	Intra-cardiac model, tail vein injection and orthotopic model	[51]
	Myosin-9	MDA-MB-231	Subcutaneous xenograft, orthotopic model	[52]
	MMPs	MDA-MB-231	Tail vein injection, orthotopic model	[53]
	MMP-1	MDA-MB-231	Tail vein injection	[54]
	NDPK	MDA-MB-231	Tail vein injection	[55]
	Annexin II	MDA-MB-231, MDA-MB-831, MDA-MB-4175	Tail vein injection and intra-cardiac model	[56]
Liver	miR-4443	MCF-7, MDA-MB-231	Orthotopic model	[57]
	miR-197	MBA-MB-231 or SUM149PT	Subcutaneous injection and tail vein injection	[58]
	E-cadherin, p120-catenin	MTPa	Orthotopic model (Rat)	[59]
	TGFβ1	MCF7, MDA-MB-231	In vitro	[60]
Brain	miR-181c	MDA-MB-231	Intra-cardiac model	[61]
	lnc GS1-600G8.5	MDA-MB-231	Intra-cardiac model	[62]
	CEMIP	MDA-MB-231	Intra-cardiac model, intracranial injection and orthotopic model	[63]
	miR-503	MCF7, ZR75-1, SKBR3, MDA-MB-231	Intra-cardiac model and intracranial injection	[64]
	miR-301a-3p	MDA-MB-231	Retro-orbital injection of EVs	[65]

## Data Availability

Not applicable.
